# Metabolomics Based Biomarkers of Green Beans Intake

**DOI:** 10.1002/mnfr.70625

**Published:** 2026-10-12

**Authors:** Rebecca Finlay, Ya Gao, Qi Sun, Frank Hu, Jayne Woodside, Xiaofei Yin, Lorraine Brennan

**Affiliations:** ^1^ UCD School of Agriculture and Food Science Institute of Food and Health University College Dublin Dublin Ireland; ^2^ UCD Conway Institute University College Dublin Dublin Ireland; ^3^ Department of Nutrition and Epidemiology Harvard T.H. Chan School of Public Health Boston Massachusetts USA; ^4^ Channing Division of Network Medicine Department of Medicine Brigham and Women's Hospital and Harvard Medical School Boston Massachusetts USA; ^5^ Centre For Public Health Queens University Belfast Belfast UK

**Keywords:** biomarkers, legumes, metabolites, metabolomics

## Abstract

While there is considerable interest in legumes as a food source, there are no validated biomarkers for green beans (*Phaseolus vulgaris*, L.) intake. The objective of the current work is to identify and assess the dose–response of food intake biomarkers of green beans. In an acute intervention study, 27 participants consumed green beans in the study center and urine samples were collected pre‐ and post‐consumption. Metabolites were measured by nuclear magnetic resonance (^1^H‐NMR) and liquid chromatography–mass spectrometry (LC–MS). Statistical analysis determined features that changed post‐consumption of green beans. Selected metabolites were assessed for dose–response in a follow‐up study (*n* = 16). Analysis revealed 745 ^1^H‐NMR spectral regions and 318 LC–MS features that increased in intensity over time following consumption of green beans. Three ^1^H‐NMR and 12 LC–MS metabolites displayed a dose–response relationship to increasing portion size of green beans (*p* <0.05), including amino acid conjugates (glycine conjugate, N‐phenylacetylglutamine), hydroxyabscisic acid glucuronide and amino‐hydroxybenzoic acid. Partial least squares discriminant analysis predicted low or high green bean intake with 91.6% accuracy. We identified 15 urinary food intake biomarkers of green beans. A biomarker panel classified individuals as low or high green bean consumers with good accuracy, progressing ongoing efforts to improve dietary assessment methods.

## Graphical abstract

Identification of urinary food intake biomarkers of green beans using metabolomics applied to time response and dose response studies.This image was created with BioRender.com.

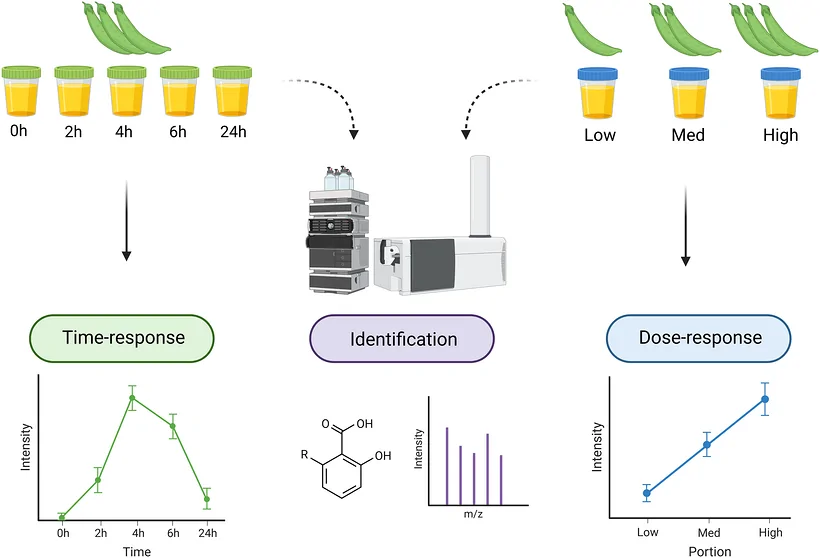

## 1 Introduction

It is now well established that diet is a major modifiable risk factor for the development of several noncommunicable diseases [1, 2, 3, 4]. Healthy dietary patterns such as the Mediterranean diet reduce the risk of developing type 2 diabetes (T2D) and cardiovascular disease (CVD) [4, 5], while the dietary approaches to stop hypertension (DASH) diet is associated with lower blood pressure [6, 7]. A common recommendation in healthy diets is high intakes of legumes, such as peas, beans, lentils, soybeans, chickpeas, and peanuts. Legumes provide vegetable protein, complex carbohydrates, micronutrients, and phytochemicals [8]. A high consumption of legumes is associated with a reduced risk of developing chronic diseases such as T2D [9, 10, 11], CVD [12, 13, 14], cancer [15, 16, 17], and osteoporosis [18, 19, 20].

Given the benefits of consuming legumes, it is essential to accurately assess legume intake at an individual or population level. However, obtaining such accuracies is challenging with traditional methods of dietary assessment, such as food diaries, 24‐h recalls, and food frequency questionnaires. There are several well documented limitations to these methods including misreporting, participant burden, difficulties estimating portion sizes, and recall bias [21, 22]. Alternatively, food intake biomarkers are a promising tool for more objective dietary assessment. Food intake biomarkers are metabolites, alone or in combination, that indicate the intake of a specific food or food group and have both a time–response and dose–response following intake of the food [23]. Through the application of metabolomic techniques to biomarker research, there are now numerous candidate biomarkers for a range of foods [24, 25, 26], including grapes [27], wholegrains [28], coffee [29], and citrus fruits [30].

There are several proposed food intake biomarkers for legumes. The isoflavones genistein and daidzein are strong candidate biomarkers for soybean intake, as each displayed a dose–response relationship and served as good estimates of long‐term intake [31]. Daidzein and its metabolites were also correlated (*r* > 0.4) to soy intakes in a 2‐week controlled feeding trial [32]. Urinary trigonelline, a frequently reported biomarker of legumes, increased in a dose‐dependent manner following increased intakes of pea during a food challenge [33]. This alkaloid was also reported to increase in urine following consumption of mixed pulses in both acute and long‐term (8‐weeks) dietary interventions [34, 35]. Serum pipecolic acid and s‐methylcysteine were reported as useful biomarkers that could separate low and high dry bean consumers [36]. Notably, these serum metabolites are frequently reported as potential biomarkers of legumes in the literature: previous works linked them to navy beans, cowpea, and white beans [37, 38, 39]. Other markers linked to pea intake include asparaginyl‐valine, N‐carbamoyl‐2‐amino‐2‐(4‐hydroxyphenyl) acetic acid, and 2‐isopropylmalic acid [40]. Sulfated compounds also feature as potential biomarkers of legume intake, such as 4‐vinylphenol sulfate for peanuts and dopamine sulfate for pulses [39, 41]. Consumption of lentils also increased urinary excretion of flavonoid‐related epicatechin metabolites and its microbial derivatives [39]. While there are emerging biomarkers in relation to legumes, there is a lack of food intake biomarkers for green beans (*Phaseolus vulgaris*, L.), the unripe pods of common beans, despite it being an important vegetable, within the legume family, with many health benefits.

The objective of this paper is to identify and assess the dose–response of food intake biomarkers of green beans through feeding trials and an untargeted metabolomics approach. Identification of such biomarkers can help in moving toward objective measures of food intake thus reducing the reliance on self‐reported data.

## 2 Experimental Section

### 2.1 Discovery Study

Ethical approval for the acute feeding study was obtained from The Human Research Ethics Committee at University College Dublin (UCD) (LS‐19‐57‐Brennan). This study aimed to identify food intake biomarkers of three foods: green beans, mixed berries (strawberries and blueberries), and mixed vegetables (spinach and lettuce). This paper, however, is focused on the identification of food intake biomarkers of green beans and the other two foods are used as control foods to address the specificity of the biomarkers. Based on previous literature of similar biomarker‐focused dietary intervention studies, a sample size of *n* = 27 was the target for the present study [27, 42]. The recruitment and screening numbers are presented in Figure S1. The inclusion criteria were healthy adults between the ages of 18 and 50 years, a body mass index (BMI) between 18.5 and 30 kg/m^2^, non‐smokers, not pregnant or lactating, not taking any medication (oral contraceptive pill was allowed), and not having any diagnosed medical condition. Interested individuals attended a screening visit and the eligible candidates were asked to sign informed consent before commencing the study. All consenting participants were randomized to consume the three test foods (green beans, mixed berries, and mixed vegetables) in a random order with a washout period of one week between each visit.

In the 24‐h before the study visit, participants were instructed to avoid consuming alcohol, medications, and any foods related to green beans. The morning of the study visit, following an overnight fast of 12‐h, participants collected a fasting first void urine sample at home and brought it to the test center in a cooler bag on ice. A large portion (240 g) of cooked green beans was consumed with 100 mL of water. Urine samples were collected at 2, 4, and 6‐h following consumption of green beans. No other foods were consumed during this 6‐h period, but 100 mL of water was provided every 2 h. Following the 6‐h urine collection, participants resumed their usual diet but continued to avoid alcohol, medication, and similar foods until the next fasting first void urine sample collection the next morning (24‐h). Urine samples were centrifuged at 1800 x *g* for 10 min at 4°C and 1 mL aliquots were stored at −80°C until further analysis.

### 2.2 Dose–Response Study

Ethical approval for the dose–response study was granted by The Human Research Ethics Committee at UCD (LS‐20‐24‐Brennan). Based on previous literature of intervention studies incorporating different portions of the test food, a sample size of *n* = 34 was the target for this dose–response study [43]. Inclusion criteria were the same as described above. The recruitment and screening numbers for this study are displayed in Figure S2. Participants were randomized into two groups: (1) green beans (*n* = 16) or (2) mixed berries (strawberries and blueberries), and spinach (*n* = 17). Each group consumed a low, medium, and high portion of the test food over 3 weeks in random order in a cross‐over design. The three portion sizes of green beans were 27, 108, and 169 g, determined from the FSA Photographic Atlas of Food Portion Sizes [44]. For each portion, participants consumed the food with their evening meal for 4 consecutive days and collected a fasting first void urine sample on the 5^th^ day. All test foods were provided by the researchers and participants used their own weighing scales to measure the correct portion of green beans each evening and boiled them for 5 minutes. To monitor adherence, volunteers sent photographs of their plates before and after consuming their evening meal with the test food. During the 4‐day testing period, participants were asked to avoid consuming any foods related to their test food. A 24‐h recall was performed with the participants on day 5 to assess adherence with food avoidance. For 24‐h prior to sample collection on day 5, participants were also instructed to avoid consuming alcohol and medications. Urine samples were centrifuged at 1800 x *g* for 10 min at 4°C and 1 mL aliquots were stored at −0°C until further analysis.

### 2.3 Metabolomic Analysis of Urine Samples

A total of five urine samples were collected per participant for each test food consumed in the discovery study, while one urine sample was collected per participant for each of the three portion sizes consumed in the dose–response study. Urine samples were analyzed by ^1^H‐NMR spectroscopy and LC–MS to cover a wide range of metabolites. For each study, samples were randomized over batches with each batch containing all samples from the same individual. Samples were prepared for ^1^H‐NMR analysis by first adding 500 µL of the thawed urine samples to 250 µL of phosphate buffer (0.2 mol KH_2_PO_4_/L, 0.8 mol K_2_HPO_4_/L). Samples were centrifuged at 5360 x *g* for 5 min at 4°C, then 540 µL of the supernatant was added to 10 µL sodium trimethylsilyl [2,2,3,3‐2H4]proprionate (TSP), and 60 µL deuterium oxide (D_2_O). Spectra were acquired on a 600 MHz Varian Spectrometer (Varian Limited, Oxford, United Kingdom) by using the first increment of a nuclear Overhauser enhancement spectroscopy pulse sequence at 25°C. Spectra were acquired with 16 384 data points and 128 scans. Water suppression was achieved during the relaxation delay (2.5s) and the mixing time (100 ms). All ^1^H‐NMR urine spectra were referenced to TSP at 0.0 parts per million (ppm) and processed manually with the Chenomx NMR Suite (Version 8.3) by using a line broadening of 0.2 Hz, followed by phase and baseline correction. Data were normalized to the total area of the spectra integral. A total of 7500 spectral regions with a bin width of 0.001 ppm were exported and the water region (4.0–6.0 ppm) was excluded. Urine metabolites were identified using Chenomx NMR suite software.

For LC–MS analysis, the urine samples were thawed on a roller and centrifuged at 5500 × *g* for 5 min at 4°C. Samples were prepared by addition of 100 µL of urine to 100 µL of the internal standards (malic acid d_3_, methionine d_3_, myristic acid ^13^C, adipic acid d_4_, and succinic acid d_4_ 10 µg/mL in 20% v/v EtOH/Millipore H_2_O). Samples were vortexed for 10 s and centrifuged at 2000 x *g* for 2 min. The supernatants were collected into vials. The samples were analyzed by an Agilent LC‐QTOF‐MS, consisting of a 1290 Infinity II LC system and an Agilent Jetstream (AJS) Electrospray ionization (ESI) source coupled to a 6545 QTOF mass spectrometer in both positive and negative ionization modes. Zorbax eclipse plus C18 2.1 × 5 mm 1.8 µm was used as a guard column and Zorbax eclipse plus C18 (2.1 × 50 mm, 1.8 µm) column was used for separation. The mobile phase was 0.1% formic acid in water (Eluent A) and 0.1% formic acid in acetonitrile/water (80:20) (Eluent B). The gradient conditions were 1% B (0–1.5 min), 11% B (1.5–9 min), 25% B (9–15 min), 50% B (15–18 min), 99% B (18–18.05 min), 99% B (18.05–21 min), 1% B (21–21.05 min), and 1% B (21.05–23 min). The osmolality of samples was determined by an Advanced Instruments Micro Osmometer model 3300 using freezing point depression. The urinary feature (metabolite) levels were normalized to osmolality.

Data were acquired using MassHunter acquisition B.08.00 software (B.08.00.8058.3 Sp1 Agilent Technologies) and were processed using MassHunter Qualitative Analysis (B.07.00 Sp2 Agilent Technologies) software. The molecular feature extractor (MFE) algorithm was used to extract molecular features characterized by retention time (RT) and *m/z* values which represent different adducts or isotopes of the same compound and provide details of the signal intensity and accurate mass. The target data type was set to small molecules and the threshold value for peak height was set to 5000 counts. The isotopic peak spacing tolerance was 0.0025 *m/z* with maximum charge state of 1 and isotope model was set to common organic molecules. The data output was then converted to Compound Exchange Format (.cef) files, which were imported into MassProfiler (Version B.07.01; Agilent technologies) for compound alignment and data filtration. The alignment of RT and m/z values was set using a tolerance of 0.5 min and 2.0mDa, respectively. The sample occurrence frequency was set to >40% across all samples. Missing values were replaced with half the minimum abundance value for that feature. Extracted ion chromatogram (EIC) data were obtained for a subset of statistically significant features.

Interesting features were initially identified by putative formula generation using MassHunter ID Browser (B 7.0.799.2 Agilent Technologies), and these formulas were compared against a local METLIN database. These features were further analyzed by MS/MS to obtain structural information of the compounds. The LC–MS/MS was performed under the same chromatographic conditions and at 3 collision energies 10, 20, and 40 eV. The compound fragmentation patterns were compared with spectral databases including METLIN, competitive fragmentation modelling for metabolite identification (CFM‐ID), and the Human metabolome database (HMDB), and the literature. Each metabolite was assigned one of five possible levels of confidence in identification: Level 0 (unambiguous structure); Level 1 (confident structure using reference standard); Level 2 (probable structure, matched to database or literature data); Level 3 (possible structure or class); and Level 4 (unknown) [45].

### 2.4 Statistical Analysis

One‐way repeated measures analysis of variance (ANOVA) was performed on the ^1^H‐NMR and LC–MS datasets from the discovery study to determine features changing over time. An FDR‐adjusted *p*‐value of <0.05 was considered statistically significant. The dose‐response data was assessed using one‐way repeated measures ANOVA analysis. The EIC data were log transformed prior to analysis. A *p*‐value <0.05 was used to indicate statistical significance. The β‐weighted z‐scores of the metabolites with a significant dose‐response were used to build partial least squares discriminant analysis (PLS‐DA) models to classify individuals as either low or high green bean consumers. The training set contained 2/3 of the data (10 low and 11 high samples), while the test set was the remaining 1/3 of the data (4 low and 4 high samples). Univariate statistical analysis was performed in Metaboanalyst 4.0 (https://www.metaboanalyst.ca/) and SPSS Statistics Version 27. Multivariate statistical analysis was performed in SIMCA 13 (SIMCA Version 13.0.3 Umetrics AB). ROC analysis was performed in Metaboanalyst 6.0 (https://www.metaboanalyst.ca/)

## 3 Results

### 3.1 Population Characteristics

In total, there were 27 participants (14 males and 13 females) enrolled on the discovery study. Of these, 26 individuals completed the green beans study visit. The discovery study participants had an average age of 23.7 ± 6.8 years and average BMI of 23.7 ± 2.8 kg/m^2^. Sixteen participants consumed green beans in the dose–response study (7 males and 9 females): the participants had an average age of 29.8 ± 9.6 years and average BMI of 23.8 ± 3.2 kg/m^2^. For the dose–response study, viable samples from all three portion sizes were obtained from 14 participants.

### 3.2 Identification of Features Related to Green Beans Intake

Analysis of the ^1^H‐NMR data from the discovery study identified 1737 spectral regions with a significant difference across time points (FDR‐adjusted <0.05). The time–response over the 6 h of all statistically significant spectral regions were examined and resulted in 745 regions that increased in intensity following green beans consumption. These were analyzed for a response to increased consumption in the dose‐response study. Examination of the ^1^H‐NMR features revealed 14 spectral regions with a significant difference across low (27 g), medium (108 g), and high (169 g) portions of green beans (*p* <0.05) (Table 1).

**TABLE 1:** ^1^H‐NMR spectral regions of interest with significant dose–response to increasing green beans consumption.

Spectral region (ppm)	Potential Identification	*p*‐value
7.3385	N‐Phenylacetylglutamine	0.016
2.3315	Unknown	0.023
2.2585	4‐Guanidinobutanoate	0.026
7.3395	N‐Phenylacetylglutamine	0.031
2.2575	4‐Guanidinobutanoate	0.034
7.4135	N‐Phenylacetylglutamine	0.036
7.4015	N‐Phenylacetylglutamine	0.037
7.3515	N‐Phenylacetylglutamine	0.039
7.3505	N‐Phenylacetylglutamine	0.042
7.3375	N‐Phenylacetylglutamine	0.044
2.2455	4‐Guanidinobutanoate	0.044
7.3495	N‐Phenylacetylglutamine	0.046
2.2445	4‐Guanidinobutanoate	0.048
7.3485	N‐Phenylacetylglutamine	0.049

^1^H‐NMR spectral regions ordered by *p*‐value from one‐way repeated measures ANOVA. A metabolite may have multiple spectral regions depending on the molecular structure. ppm, parts per million.

For the LC–MS data, 713 positive mode and 908 negative mode features were obtained from urine samples after consumption of green beans. A total of 637 features (226 positive and 411 negative) were significantly different across time points (FDR‐adjusted <0.05). Following examination of the time‐response data, 98 positive mode and 220 negative mode features had increased in intensity post‐consumption of green beans (Figure 1). Analysis of the dose–response data resulted in 26 features (10 positive and 16 negative mode) with a significant difference in peak height across low, medium, and high portions (*p* <0.05) (Table S1). EIC data was obtained for these features and further analysis revealed a significant dose‐response for 15 features (Figure 2), where 13 of these increased across the different portion sizes (Figure 2: A–H, K–O). One metabolite appeared in both ionization modes; thus, the 13 features correspond to 12 metabolites. Finally, these 12 metabolites were included in the green beans biomarker panel to classify low and high consumers.

**FIGURE 1:**
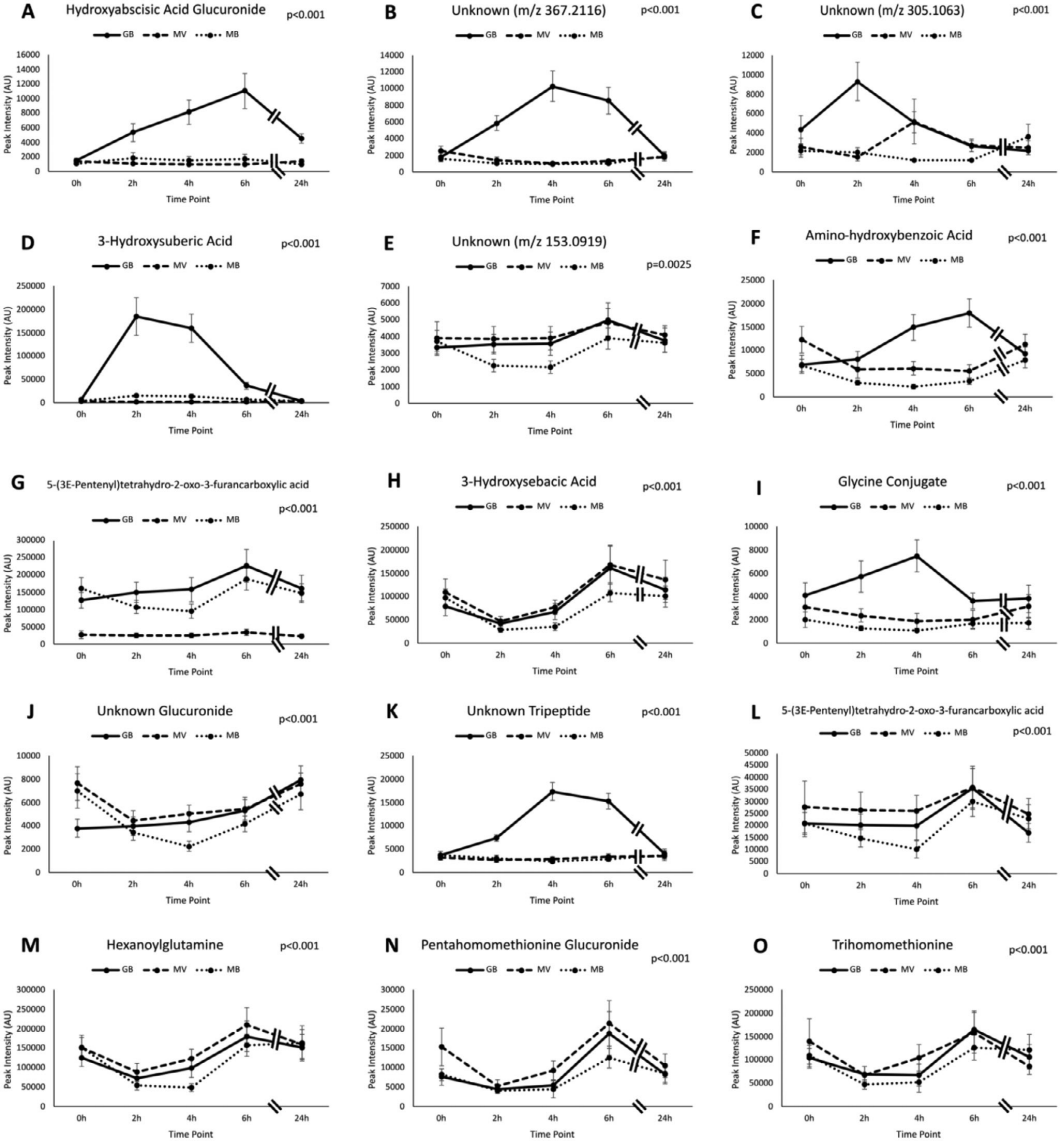
Selection of metabolites with a significant time response in urine samples following consumption of green beans (FDR‐adjusted <0.05). Data are from the discovery study. Values are means ± SEM (standard error of the mean). (A–J) metabolites found in negative mode. (K–O) metabolites found in positive mode. GB: green beans (solid line); MV: mixed vegetables (dashed line); MB: mixed berries (dotted line); AU: arbitrary units; 0 h: baseline; 2 h: 2‐hours; 4 h: 4‐hours; 6 h: 6‐hours; 24 h: 24‐hours.

**FIGURE 2:**
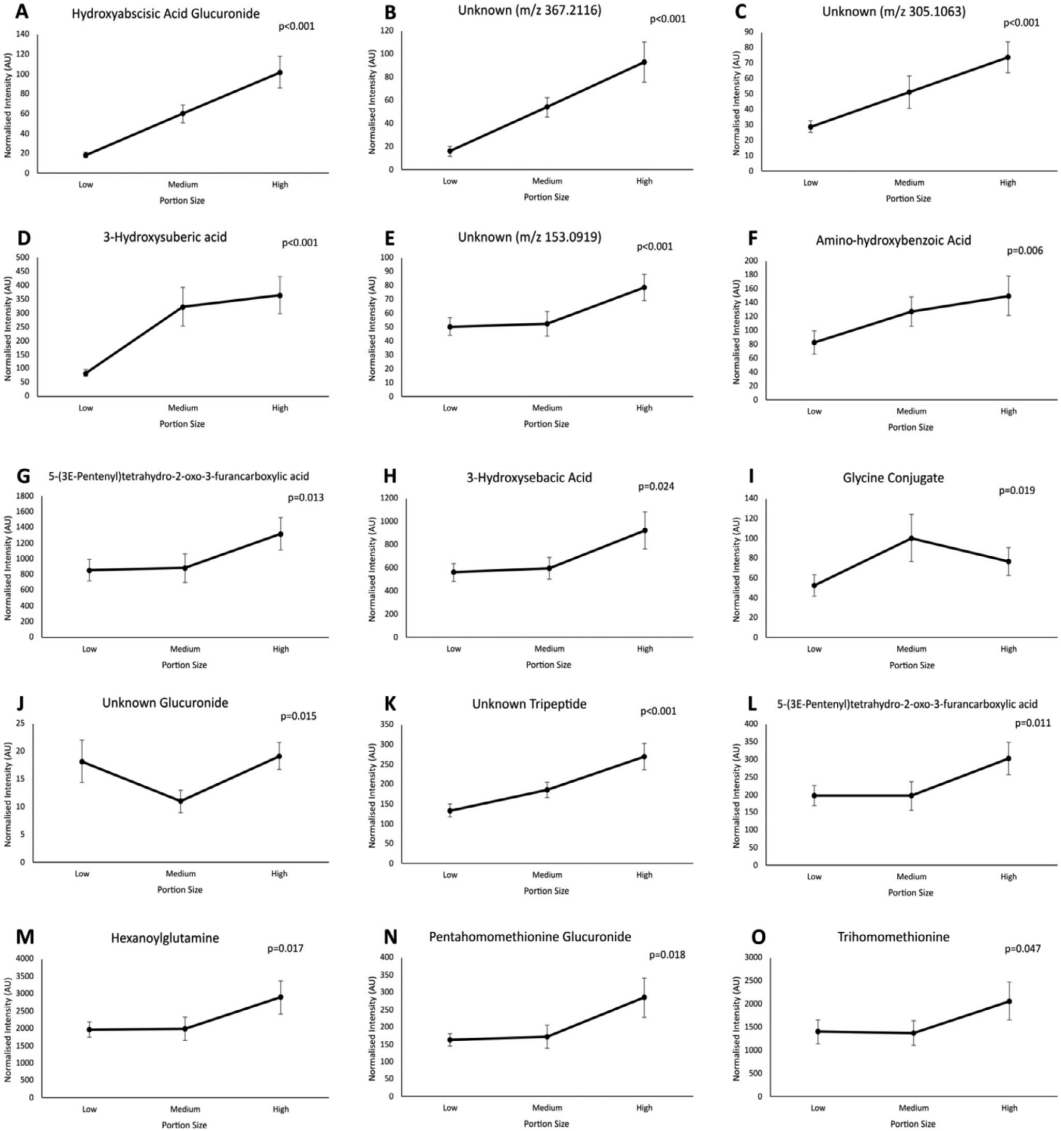
Extracted ion chromatogram (EIC) data of metabolites with a significant difference across low, medium and high intakes of green beans (*p*<0.05). Values are means ± SEMs. AU: arbitrary units.

### 3.3 Biomarkers Reflecting Green Beans Intake

The 14 spectral regions from the ^1^H‐NMR data with a significant dose‐response to green beans intake correspond to three metabolites (Table 1) For the LC–MS data, level 2 identification was obtained for 8 of the 13 significant features, with the remaining 7 assigned a compound class (level 3) or were unknown (level 4) (Table 2).

**TABLE 2:** Putative identifications for interesting features displaying a dose‐response to green beans intake.

RT	Mass	m/z	Ion	MS/MS	Putative identification	Putative formula	*p*‐value
1.551	273.1686	274.1758	[M+H]+	85.0285, 100.112, 114.0554, 125.071	Unknown Tripeptide (3)	C12 H23 N3 O4	<0.001
4.668	153.0425	152.0354	[M‐H]‐	51.0242, 80.0505, 107.0371, 108.045, 109.0296	An amino‐hydroxybenzoic acid isomer (3)	C7 H7 N O3	0.006
9.265	191.0982	192.1053	[M+H]+	61.0104, 69.0698, 97.0645, 115.0753, 175.0786	Trihomomethionine (2)	C8 H17 N O2 S	0.047
11.054	244.1424	245.1496	[M+H]+	71.0853, 84.0444, 129.0657, 130.0499, 147.0764	Hexanoylglutamine (2)	C11 H20 N2 O4	0.017
11.314	190.0838	189.0767	[M‐H]‐	41.003, 57.0346, 99.0815, 127.0762, 129.0559	3‐Hydroxysuberic acid (2)	C8 H14 O5	<0.001
12.32	306.113	305.1063	[M‐H]‐	59.0137, 79.9574, 106.9806, 113.024, 124.0066, 132.0448, 175.023, 308.1144	Unknown (4)	C12 H16 N7 O S	<0.001
12.573	456.1616	455.154	[M‐H]‐	59.0141, 71.0138, 85.0293, 96.9597, 113.0238, 151.0756, 175.0238, 217.1213, 279.1209	Hydroxyabscisic acid glucuronide (2)	C21H28O11	<0.001
13.445	242.1147	241.1076	[M‐H]‐	59.0139, 74.0247, 165.055, 181.0859, 197.117, 204.0655, 223.0963	Glycine conjugate (3)	—	0.019
13.532	218.115	217.1079	[M‐H]‐	59.0137, 155.1071, 157.0863, 171.1017	3‐Hydroxysebacic acid (2)	C10 H18 O5	0.023
14.663	154.099	153.0919	[M‐H]‐	59.0142, 83.0501, 111.0811, 135.0803	Unknown (4)	C9 H14 O2	0.006
14.664	198.0892	197.082	[M‐H]‐	41.003, 59.0137, 83.0502, 111.0449, 135.0814, 153.0902	5‐(3E‐Pentenyl)tetrahydro‐2‐oxo‐3‐furancarboxylic acid (2)	C10 H14 O4	0.013
14.787	198.0892	199.0965	[M+H]+	55.0174, 77.0381, 91.0539, 111.0438, 135.0798, 153.0905, 181.0855	5‐(3E‐Pentenyl)tetrahydro‐2‐oxo‐3‐furancarboxylic acid (2)	C10 H14 O4	0.011
14.993	395.1613	396.1688	[M+H]+	97.1002, 125.0949, 143.1053, 203.109, 220.1355	Pentahomomethionine glucuronide (2)	C16 H29 N O8 S	0.018
15.436	306.1337	305.1264	[M‐H]‐	59.0138, 85.029, 113.024, 129.0914, 145.0605, 175.0232, 219.1048, 269.1474	Unknown Glucuronide (4)	C13H22O8	0.015
16.19	368.2186	367.2116	[M‐H]‐	125.0239, 220.0644, 285.1856, 303.1963	Unknown (4)	C20 H32 O6	<0.001

Abbreviations: RT, retention time; m/z, mass‐to‐charge ratio; MS/MS, tandem mass spectrometry main fragments; *p*‐values are from one‐way repeated measures ANOVA using EIC data from dose–response study samples. Numbers next to identification indicate confidence level.

3‐Hydroxysuberic acid with *m/z* 189.0767 was identified in negative ionization mode. Using CFM‐ID, the inputted spectra matched well with the 3‐hydroxysuberic acid predicted spectra from the HMDB database with a similarity score of 0.6434. The low collision energy (6 eV) experimental MS/MS spectrum from the HMDB database also matched our 10‐eV spectrum in terms of the main fragments at *m/z* 129.056, 127.073, and 99.0832 and fragment intensities. Another interesting metabolite is hydroxyabscisic acid glucuronide with *m/z* 455.154. The MS/MS spectra for this metabolite displayed a neutral loss of ≈176.03 Da from the parent ion to the fragment ion *m/z* 279.1213, indicating the loss of a glucuronide (C6 H8 O6). The glucuronide moiety fragments *m/z* 175.0235 and 113.024 were also present in the spectra. The remaining fragments matched well to the predicted MS/MS spectra for 13‐hydroxyabscisic acid in HMDB including m/z 261.11, 235.13, 217.12, and 205.12. In addition, all major fragments for this metabolite matched well with those reported previously for hydroxyabscisic acid glucuronide [46]. 3‐Hydroxysebacic acid appeared in negative ionization mode with m/z 217.1079. The MS/MS spectra matched the predicted spectra from HMDB with a similarity score of 0.7659. The low collision energy spectra also matched an experimental spectrum (6 eV) for 3‐hydroxysebacic acid from the Massbank database in terms of the main fragments at m/z 171.099, 172.10, and 199.09.

The three PLS‐DA models built using the β‐weighted z‐scores of the metabolites had Q^2^ values greater than 0.4 (Table 3). The *R* ^2^ values for these PLS‐DA models ranged from 0.70 to 0.82. The average correct classification rate of the three test sets was 91.6%. In both cases, the misclassified samples were from the high green beans portion group. ROC analysis revealed that the highest AUC (0.95) was obtained from a model with three metabolites: 3‐Hydroxysuberic acid, hydroxyabscisic acid glucuronide and an unknown.

**TABLE 3:** Correct classification rates for PLS‐DA test sets.

Test Set	*Q* ^2^	Correct predictions	Percentage
1	0.448	7/8	87.5%
2	0.408	7/8	87.5%
3	0.405	8/8	100%
Total		22/24	91.6%

*Q* ^2^: the predictive ability of the PLS‐DA model. The 12 unique metabolites displaying a positive dose‐response to increasing green bean intakes were used to generate the PLS‐DA models. Data were the β‐weighted z‐scores of osmolality normalized metabolite levels. The predications represent the number of participants that were correctly classified as low or high green bean consumers, based on urinary metabolite data.

## 4 Discussion

The present study identified several urinary biomarkers displaying a dose–response relationship to green beans intake. The putative biomarkers also increased in urine following green beans intake in an acute setting. The robustness of the biomarkers is confirmed by the fact that the dose–response study was performed when participants consumed their habitual diet. Furthermore, the biomarker panel was capable of classifying individuals as low or high green bean consumers with good accuracy.

The current research used a multi‐platform untargeted metabolomics approach to identify a panel of food intake biomarkers for green beans. One of the nine criteria for the validation of food intake biomarkers previously proposed is biological plausibility [23, 47]. Several of the proposed biomarkers in this study have high plausibility, including 3‐hydroxysuberic acid, hydroxyabscisic acid glucuronide, and the amino‐hydroxybenzoic acid isomer. 3‐Hydroxysuberic acid is a medium chain hydroxy acid that is functionally related to suberic acid. Suberic acid is reported to be present in common bean (*Phaseolus vulgaris*) [48] and the leaves of pea (*Pisum savitum*) [49]. In addition, the chemical formula and MS/MS spectra for 3‐hydroxysuberic acid in the present study matched with those of a previously reported urinary metabolite found after consumption of green beans [50]. Hydroxyabscisic acid glucuronide is a plausible biomarker for green bean intake as the 13‐hydroxyabscisic acid isomer was reported in cowpea [51]. Hydroxyabscisic acid glucuronide was also identified in urine following ingestion of a bilberry supplement, but this is likely to be the 8‐hydroxyabscisic acid isomer which was previously linked to berries and other fruits [52]. In the current study, urinary excretion of hydroxyabscisic acid glucuronide significantly increased after consumption of green beans, whereas there was minimal change after intake of mixed berries. Although it was not fully identified, another noteworthy metabolite is the amino‐hydroxybenzoic acid, as green beans are known to contain various phenolic acids including hydroxybenzoic acid derivatives [53, 54]. This metabolite exhibited similar MS/MS spectra to multiple amino‐hydroxybenzoic acid isomers, however, no specific identification could be assigned. One possible isomer is 3‐hydroxyanthranilic acid (also known as 2‐amino‐3‐hydroxybenzoic acid), which was isolated from tempeh, a food made from fermented soybeans [55]. Information on the other isomers in relation to their natural origins is lacking. More research is needed to identify this metabolite in urine of individuals following consumption of green beans or other legumes and to assign a higher‐level identification. Overall, these three metabolites are a good addition to the biomarker panel as they have high biological plausibility as biomarkers of green beans intake.

There are some previously reported biomarkers for legumes that were not identified in the present study. Most studies investigating biomarkers of legumes were focused on soybeans, with both blood and urinary genistein and daidzein frequently reported after intake of soybeans or soybean products [31, 56]. Unsurprisingly, these isoflavones were not found in the present study as they are considered highly specific to soybeans. This can be explained by the very high concentrations of genistein and daidzein in soybeans compared to green beans and other legumes [56]. In the current study, urine was the biofluid used for biomarker identification as it is known to contain many food‐derived metabolites and it is noninvasive [57, 58]. For this reason, previously proposed serum biomarkers such as pipecolic acid, s‐methylcysteine, tryptophan betaine, and 4‐vinylphenol sulfate did not feature in the urinary panel. The alkaloid trigonelline is often reported in urine following intake of various legumes [33, 34, 35, 36]. Interestingly, in the present study trigonelline levels increased in post‐consumption samples, but the increase from baseline was not significant. Some varieties of common bean (*Phaseolus vulgaris*) and other legumes are known to contain trigonelline [34, 59], but perhaps the concentration in green beans is too low to produce a significant increase in urine following consumption. Other previously reported biomarkers for legumes include amino acids and other nitrogen‐containing compounds such as glutamine, lysine, dimethylglycine, dimethylamine, trimethylamine, and 3‐methylhistidine [34, 60]. While the present study did not find these exact metabolites, there are multiple amino acid conjugates and derivatives in the biomarker panel including 4‐guanidinobutanoate, N‐phenylacetylglutamine, hexanoylglutamine, a glycine conjugate, trihomomethionine, pentahomomethionine glucuronide, and an unknown tripeptide. These amino acid‐related compounds are not specific to green beans; however, they may be useful in combination to provide a more accurate estimate of intake.

Recent research has highlighted the need for panels of biomarkers to capture food intake [61, 62, 63, 64]. A panel of 16 biomarkers for coffee consumption performed well at discriminating between coffee consumers and non‐consumers, with an average area under the receiver operating characteristic curve of 0.98 [61]. Interestingly, different combinations of just two to five of these biomarkers performed even better. Similarly, combinations of two or three biomarkers of red meat intake were better at classifying individuals than each biomarker alone or all together, with carnosine, creatine or N‐(1‐methyl‐4‐hydroxy‐3 imidazolin‐2,2‐ylidene)alanine included in the best performing models [63]. Additionally, multi‐metabolite models were built to predict cranberry juice consumption using either plasma or urine metabolites [65, 66]. The 29‐plasma metabolite model could correctly classify cranberry juice consumers with 93.8% accuracy, while the urine metabolite models predicted consumers with between 84% and 100% accuracy. The current green beans model had similar accuracies when predicting low and high green bean consumers with an overall correct prediction rate of 91.6%. Moving beyond classification of consumers, a latent variable model composed of eight metabolites, including s‐methyl cysteine sulfoxide, was used to predict intake of broccoli in grams from biomarker data alone [67]. The combined model had good agreement for low (73%) and medium (80%) intakes of broccoli. The addition of our work to this growing body of evidence highlights the potential of multi‐biomarker panels to accurately estimate food intake and improve dietary assessment.

In the same regard, plasma metabolomic signatures were identified for single foods or food groups, including legumes, olive oils, dairy, and meat, which had good correlations to reported intakes [68, 69, 70, 71]. The legume signature consisted of 40 metabolites, both positively and negatively correlated, with the strongest positive correlations for N‐acetylornithine, C16:1 sphingomyelin, lactate, homoarginine, C18:2 carnitine, and N‐acetylaspartic acid [68]. Importantly, this signature was associated with reduced risk of T2D incidence in an older Mediterranean population. In addition, several metabolomic signatures, mainly comprised of lipid metabolites, amino acids and xenobiotics, were developed to measure adherence to the Mediterranean diet [72, 73, 74], other healthy dietary patterns [75, 76], and overall healthy lifestyle [77, 78]. Many of these studies also investigated the signatures further for their relation to T2D and CVD risk, with metabolomic signatures of healthy dietary patterns and lifestyle consistently associated with reduced risk of disease [75, 76, 77, 78]. Previous work from our research group identified specific metabolites that were associated with higher healthful plant‐based diet (hPDI) index scores and were previously associated with lower T2D risk [79]. Similarly, in participants of the MASALA cohort, higher hPDI metabolite profile scores were associated with favorable cardiometabolic markers [80]. Collectively, these studies demonstrate the potential of metabolomic signatures or combined metabolite scores.

A limitation of the current work is the inability to assign full identifications to all metabolites in the biomarker panel. Metabolite identification is a known bottleneck in metabolomics research due to lack of authentic standard availability and limited food‐related compounds in MS/MS databases. Additionally, as the green beans were consumed at home during the dose‐response study, full adherence with the assigned portion sizes cannot be guaranteed. However, measures were taken to encourage adherence, including photographs of meals before and after consumption and a 24‐h recall on day 5. On the other hand, this study had several strengths. It combined both an acute study and a dose–response study to ensure that robust and responsive biomarkers were identified. The current work employed an untargeted approach using both ^1^H‐NMR and LC–MS techniques, facilitating the measurement of a large variety of metabolites related to green beans consumption. Finally, green beans were consumed in a controlled environment during the acute study and several measures were taken to ensure adherence within the dose–response study, while allowing participants to consume their habitual diet.

This study demonstrates the successful application of a multi‐platform untargeted metabolomics approach to identify food intake biomarkers of green beans intake. This research revealed 15 metabolites displaying both a time‐response and dose–response relationship to green beans intake. The biomarker panel demonstrated a good ability to classify individuals as low or high consumers of green beans. Future work is needed to reproduce these results and investigate the use of this biomarker panel in different population groups. This work is an important addition to current efforts in the improvement of objective dietary intake assessment methods.

## Conflicts of Interest

The authors declare no conflicts of interest.

## Supporting information




**Supporting File**: mnfr70625‐sup‐0001‐SuppMat.docx.

## Data Availability

The data that support the findings of this study are available from the corresponding author upon reasonable request. The data are not publicly available due to privacy or ethical restrictions.
