# Supplementary material for: Metabolomics Based Biomarkers of Green Beans Intake
Source: Mol Nutr Food Res. 2026 Oct 12;70(19):e70625. doi: 10.1002/mnfr.70625 (PMC13655637; doi:10.1002/mnfr.70625)
Supplement: Supplementary file 1 [file MNFR-70-e70625-s001.docx]

**Metabolomics based biomarkers of green beans intake**

Rebecca Finlay^1,2^, Ya Gao^1,2^, Qi Sun^3,4^, Frank Hu^3,4^, Jayne Woodside^5^, Xiaofei Yin^1,2^, Lorraine Brennan^1,2^

^1^ UCD School of Agriculture and Food Science, Institute of Food and Health, University College Dublin, Belfield, Dublin 4, Ireland

^2^ UCD Conway Institute, University College Dublin, Belfield, Dublin 4, Ireland

^3^ Department of Nutrition and Epidemiology, Harvard T.H. Chan School of Public Health, Boston, MA, USA.

^4^ Channing Division of Network Medicine, Department of Medicine, Brigham and Women’s Hospital and Harvard Medical School, Boston, MA, USA.

^5^ Centre for Public Health, Queens University Belfast, Belfast, United Kingdom

Supplementary Materials

Contacted (n= 64)

Excluded (n= 31)

♦  Not meeting inclusion criteria (n= 8)

♦  Declined to participate (n= 12)

♦  No response (n= 11)

Screened (n= 33)

Excluded (n= 6)

♦  Not meeting inclusion criteria (n= 1)

♦  Declined to participate (n= 4)

♦  Other reasons (n= 1)

Completed 3 visits (n= 23)

Randomised (n= 27)

Completed 2 visits (n= 2)

Completed 1 visit (n= 2)

## Allocation

Green beans:

Allocated (n= 27)

Consumed (n= 26)

Spinach & Lettuce:

Allocated (n= 27)

Consumed (n= 24)

Strawberries & Blueberries:

Allocated (n= 27)

Consumed (n= 25)

**Supplementary Figure 1:** Outline of recruitment procedure for the discovery study.

Contacted (n= 113)

Excluded (n= 64)

♦  Not meeting inclusion criteria (n= 15)

♦  Declined to participate (n= 34)

♦  No response (n= 15)

Screened (n= 49)

Excluded (n= 14)

♦  Not meeting inclusion criteria (n= 9)

♦  Declined to participate (n= 3)

♦  Other reasons (n= 2)

Randomised (n= 35)

Males (n=15)

Females (n=20)

## Allocation

Green beans:

Allocated (n= 17)

*1 dropout

Berries & Spinach:

Allocated (n= 18)

*1 dropout

Completed 3 visits (n= 15)

Completed 2 visits (n= 1)

Completed 3 visits (n= 16)

Completed 2 visits (n= 1)

Dropout (n=2)

**Supplementary Figure 2:** Outline of recruitment procedure for the dose-response study.

**Supplementary Table 1:** Putative identifications for features with a significant difference across portions of green beans intake.

| **RT** | **Mass** | **m/z** | **Ion** | **MS/MS** | **Putative Identification** | **Putative Formula** | **P-value** |
| --- | --- | --- | --- | --- | --- | --- | --- |
| 1.551 | 273.1686 | 274.1758 | [M+H]+ | 85.0285, 100.112, 114.0554, 125.071 | Unknown Tripeptide | C12 H23 N3 O4 | <0.001* |
| 1.628 | 205.9881 | 204.9815 | [M-H]- | 41.0031, 71.0502, 79.9573, 87.0088, 97.0294, 125.0244 | Phloroglucinol Sulfate | C6 H6 O6 S | 0.039 |
| 3.851 | 229.0043 | 230.0119 | [M+H]+ | 77.0384, 95.0493, 122.0603, 132.0447, 150.055, 167.0816 | 5-Hydroxy-6-indolyl-O-sulfate | C8 H7 N O5 S | 0.029 |
| 3.948 | 499.0875 | 498.0814 | [M-H]- | 227.9954, 172.9906, 148.0397, 79.9577 | Unknown Sulfate | C19 H21 N3 O11 S | 0.026* |
| 4.668 | 153.0425 | 152.0354 | [M-H]- | 51.0242, 80.0505, 107.0371, 108.045, 109.0296 | An amino-hydroxybenzoic acid isomer | C7 H7 N O3 | 0.006 |
| 5.1 | 210.0637 | 209.0563 | [M-H]- | 79.9575, 80.9653, 108.045, 132.0451, 165.0658, 212.0007 | Unknown Sulfate | C9 H10 N2 O4 | 0.046 |
| 6.317 | 188.0681 | 187.0607 | [M-H]- | 59.0139, 74.0247, 79.9571, 109.0286, 116.0348, 127.0758, 143.0707 | Nα-Acetyl-L-glutamine | C7 H12 N2 O4 | 0.007 |
| 9.055 | 513.0921 | 512.0849 | [M-H]- | 79.9575, 107.05, 145.061, 187.0059, 263.1018 | Unknown | C25 H15 N5 O8 | 0.02 |
| 9.265 | 191.0982 | 192.1053 | [M+H]+ | 55.054, 61.0104, 69.0698, 97.0645, 115.0753, 175.0786 | Trihomomethionine | C8 H17 N O2 S | 0.047 |
| 10.201 | 152.12 | 153.1272 | [M+H]+ |  | Dihydrocarvone | C10 H16 O | 0.025 |
| 11.054 | 244.1424 | 245.1496 | [M+H]+ | 71.0853, 84.0444, 129.0657, 130.0499, 147.0764 | Hexanoylglutamine | C11 H20 N2 O4 | 0.034 |
| 11.314 | 190.0838 | 189.0767 | [M-H]- | 41.003, 57.0346, 99.0815, 127.0762, 129.0559 | 3-Hydroxysuberic acid | C8 H14 O5 | <0.001 |
| 12.32 | 306.113 | 305.1063 | [M-H]- | 59.0137, 79.9574, 106.9806, 113.024, 124.0066, 132.0448, 175.023, 308.1144 | Unknown | C12 H16 N7 O S | 0.004 |
| 12.573 | 456.1616 | 455.154 | [M-H]- | 59.0141, 71.0138, 85.0293, 96.9597, 113.0238, 151.0756, 175.0238, 217.1213, 279.1209 | Hydroxyabscisic acid glucuronide | C21 H28 O11 | <0.001* |
| 13.445 | 242.1147 | 241.1076 | [M-H]- | 59.0139, 74.0247, 165.055, 179.1069, 181.0859, 197.117, 204.0655, 223.0963 | Glycine conjugate | - | 0.03* |
| 13.532 | 218.115 | 217.1079 | [M-H]- | 59.0137, 155.1071, 157.0863, 171.1017 | 3-Hydroxysebacic acid | C10 H18 O5 | 0.029 |
| 14.663 | 154.099 | 153.0919 | [M-H]- | 135.0803, 111.0811, 83.0501, 59.0142 | Unknown | C9 H14 O2 | 0.006 |
| 14.664 | 198.0892 | 197.082 | [M-H]- | 41.003, 59.0137, 83.0502, 111.0449, 135.0814, 153.0902 | 5-(3E-Pentenyl)tetrahydro-2-oxo-3-furancarboxylic acid | C10 H14 O4 | 0.01 |
| 14.787 | 198.0892 | 199.0965 | [M+H]+ | 55.0174, 77.0381, 91.0539, 111.0438, 135.0798, 153.0905, 181.0855 | 5-(3E-Pentenyl)tetrahydro-2-oxo-3-furancarboxylic acid | C10 H14 O4 | <0.001 |
| 14.993 | 395.1613 | 396.1688 | [M+H]+ | 97.1002, 125.0949, 143.1053, 203.109, 220.1355 | Pentahomomethionine glucuronide | C16 H29 N O8 S | 0.038 |
| 15.436 | 306.1337 | 305.1264 | [M-H]- | 59.0138, 85.029, 113.024, 129.0914, 145.0605, 175.0232, 219.1048, 269.1474 | Unknown glucuronide | C13 H24 O8 | 0.02* |
| 15.986 | 219.1294 | 220.1361 | [M+H]+ | 55.054, 61.0104, 83.0853, 97.1011, 125.0958, 143.1063, 203.1099 | Pentahomomethionine | C10 H21 N O2 S | 0.038 |
| 15.988 | 202.1027 | 203.1099 | [M+H]+ | 55.0539, 61.0105, 83.0851, 97.101, 107.0851, 125.0956, 143.1059 | Pentahomomethionine derivative | C10 H18 O2 S | 0.03 |
| 16.19 | 368.2186 | 367.2116 | [M-H]- | 79.9569, 125.0239, 220.0644, 285.1856, 303.1963 | Unknown | C20 H32 O6 | <0.001 |
| 16.815 | 346.0712 | 345.0651 | [M-H]- | 59.0139, 71.0139, 75.009, 79.9577, 85.0294, 113.0238, 165.0185, 180.0413, 207.0652, 250.0831, 265.1061 | 3-{3-[(3,3-dimethyloxiran-2-yl)methyl]-4-(sulfooxy)phenyl}-2-hydroxypropanoic acid | C14 H18 O8 S | 0.014 |
| 16.944 | 289.1709 | 290.1785 | [M+H]+ | 67.0541, 91.054, 111.0437, 136.0515, 154.0623, 182.0571, 197.0804 | Ubiquinone conjugate | C14 H27 N O3 S | 0.006 |

RT: retention time; m/z: mass to charge ratio; MS/MS: tandem mass spectrometry main fragments; p-values are from one-way repeated measures ANOVA; *: EIC data used where no original dose-response data could be found.
